# Overview of Research on Vanadium-Quercetin Complexes with a Historical Outline

**DOI:** 10.3390/antiox11040790

**Published:** 2022-04-17

**Authors:** Agnieszka Ścibior

**Affiliations:** Laboratory of Oxidative Stress, Centre for Interdisciplinary Research, The John Paul II Catholic University of Lublin, 20-708 Lublin, Poland; agnieszka.scibior@kul.pl

**Keywords:** vanadium, quercetin, vanadium-quercetin complexes, in vitro/in vivo studies, antidiabetic/antitumoral activity, antioxidant potential, historical framework

## Abstract

The present review was conducted to gather the available literature on some issues related to vanadium-quercetin (V-QUE) complexes. It was aimed at collecting data from in vitro and in vivo studies on the biological activity, behavior, antioxidant properties, and radical scavenging power of V-QUE complexes. The analysis of relevant findings allowed summarizing the evidence for the antidiabetic and anticarcinogenic potential of V-QUE complexes and suggested that they could serve as pharmacological agents for diabetes and cancer. These data together with other well-documented biological properties of V and QUE (common for both), which are briefly summarized in this review as well, may lay the groundwork for new therapeutic treatments and further research on a novel class of pharmaceutical molecules with better therapeutic performance. Simultaneously, the results compiled in this report point to the need for further studies on complexation of V with flavonoids to gain further insight into their behavior, identify species responsible for their physiological activity, and fully understand their mechanism of action.

## 1. Introduction

**Figure 1:**
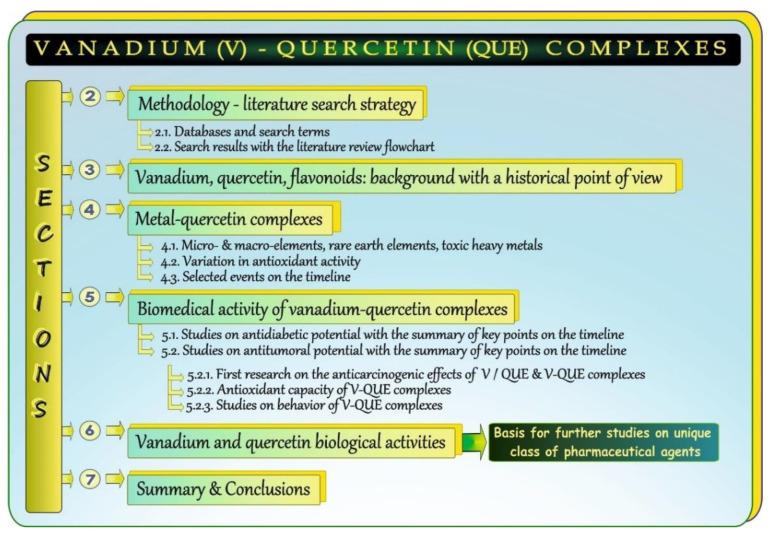
Graphical summary of the overviewed issues.

The present paper is an attempt to provide a thorough review on some issues related to complexes of vanadium (V) and quercetin (QUE). After the Introduction, the article comprises a few main sections and subsections, as illustrated in Figure 1. Section 2 introduces the reader to the methodology. It provides information about the search strategy, i.e., it shows the sources and date of searching, the keywords used to identify relevant articles, and a flowchart of the literature review process. Section 3 provides a concise summary of basic information about V and QUE and illustrates selected events related to V and QUE on the timeline. Section 4, which is composed of three subsections, Section 4.1, Section 4.2, and Section 4.3, overviews metal-QUE complexes. The first subsection summarizes data on complexes between different metals and QUE, the second one collects information about variations in their antioxidant activity, and the last one presents a brief historical background related to **the beginning of research on V-QUE** complexes with elements of the chemistry of vanadium. The next section (Section 5) consists of two subsections, Section 5.1 and Section 5.2, the latter of which is further divided into three parts, Section 5.2.1, Section 5.2.2 and Section 5.2.3. It compiles data on the biomedical activity of V-QUE complexes. More precisely, this part summarizes the first studies on the insulin-like effects and antitumoral activities of V and QUE, as well as data obtained from research on the anti-diabetic and anti-carcinogenic properties of V-QUE complexes and their behavior. Moreover, a brief outline of the antioxidant capacity of V-QUE complexes is provided. Section 6 briefly overviews the biological activities of V and QUE that are common for both V and the aforementioned flavonol. Finally, Section 7 provides a summary and conclusions.

One of the main goals of this review was to provide concise knowledge of selected properties of V-QUE complexes. Hence, the most important literature findings from both in vitro and in vivo studies of the antidiabetic and antitumoral potential of V-QUE complexes and their behavior, antioxidant capacity, and radical scavenging activity have been overviewed and illustrated in a form accessible to anyone interested in metal complexes in general. Additionally, a historical framework has been added to offer the reader an insight into certain events related to research on V, QUE, and V-QUE complexes. This type of approach not only provides more thorough knowledge on the topic but is also important from an educational point of view.

## 2. Methodology—Literature Search Strategy

### 2.1. Databases and Search Terms

The literature search in English-language databases such as PubMed (NCBI), Scopus, and Web of Science (WoS) was conducted from 29 January 2022 to 11 February 2022 to identify studies on the medicinal potential of V-QUE complexes, i.e., on their antidiabetic and antitumoral activities, behavior, and interactions with biological ligands. The search was focused on the “Title/Abstract”, and such keywords as “vanadium”, “quercetin”, “flavonoids”, “vanadyl”, “vanadium complexes”, “oxovanadium”, “cancer”, “tumor”, “antitumor”, “anticarcinogenic”, “diabetes”, “diabetic”, “insulin-mimetic”, and “antidiabetic” linked with “AND” in various combinations were used. If no full-text articles were available (only abstracts), the author of the current report contacted the corresponding author of the original papers via email. Moreover, the references lists of the selected articles were manually reviewed to search additional records (e.g., full-text papers) relevant to the topic. Additionally, certain websites were used.

### 2.2. Search Results and Literature Review Flowchart

**Figure 2:**
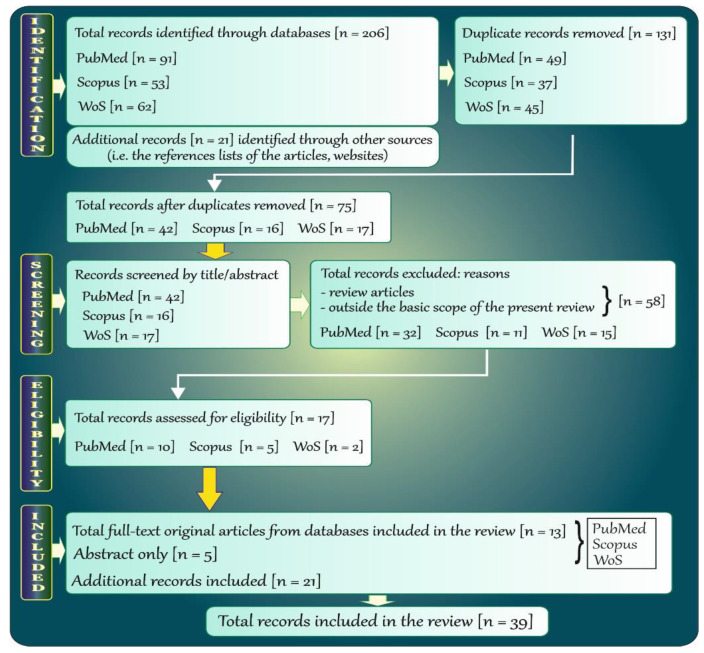
Flow chart of the systematic literature review.

The adopted strategy of searching allowed detection of records in PubMed (NCBI), Scopus, and WoS and made it possible to find some of them in other sources. The next steps followed the search. At the beginning, all duplicate records (i.e., *n* = 131) revealed by the databases were removed. Next, the remaining records (*n* = 75) were initially screened by the titles and abstracts. Afterwards, review articles along with records that deviated from the topic addressed in this report were removed (*n* = 58), and 17 potentially relevant records were further examined. Finally, a total of 13 full-text original articles in English and only 5 abstracts (identified in the databases) as well as 21 additional records were included in the current review. In the case of papers published in other languages (e.g., in German, or Japanese), only their abstracts published in English were included. A flow chart (with more details) illustrating the steps of the methodology is provided below (Figure 2). 

## 3. V, QUE, FLAV—Background with a Historical Point of View

Vanadium (chemical symbol V) is a grayish metal and one of the d-block transition elements. It is located in period 4 and Group Vb of the periodic table. It may exist in multiple oxidation states, but 5+, 4+, and 3+, i.e., vanadate (VO_3_^−^), vanadyl (VO^2+^), and vanadic (V^3+^) forms, respectively, are the most common states [1,2]. In both anionic and cationic forms, this metal may interact with a variety of biological ligands, as summarized by Rheder [1] in a review article on bioinorganic vanadium chemistry. Due to its unique features, V has received considerable attention from many scientists worldwide. Its potential therapeutic use has become a subject of special interest and was presented by the author of the current report and co-workers previously [3]. 

**Figure 3:**
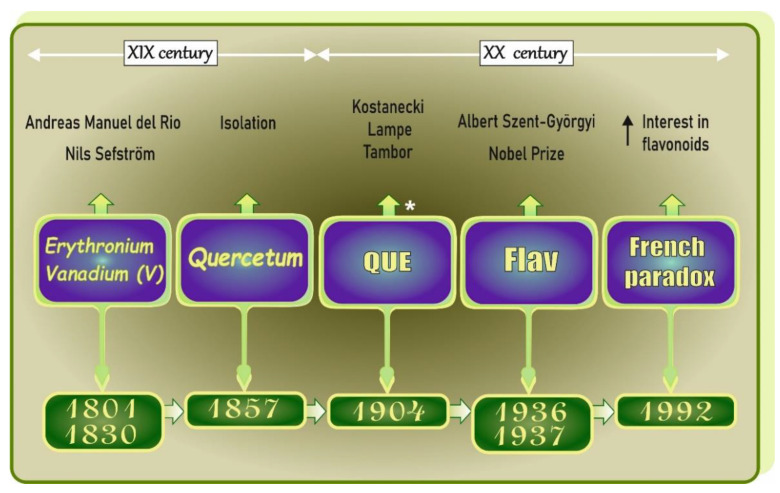
Historical view on vanadium (V), quercetin (QUE), and flavonoids (Flav). Elaborated on the basis of available literature data [4,5,6,7,8,9,10]. * after [11]. Flav: flavonoids; QUE: quercetin; V: vanadium. ↑: increase.

According to official sources (Figure 3) [4,5,6,7,8,9,10], V was discovered for the first time in 1801 by Andrés Manuel del Río (Spanish mineralogist), who named this new element “*erythronium*” from the red color of the compound [4]. However, due to the mistake made by French chemist Collett-Desotils, the discovery was disputed and, after over thirty years, i.e., in 1830, Nils Sefström (Swedish chemist) rediscovered this metal and named it “*vanadium*” in honor of the Scandinavian goddess of beauty and fertility, Vanadis (Freyja) [4].

Quercetin (QUE), 3,3′,4′,5,7-pentahydroxyflavone, is a natural flavonoid classified into the flavonol subgroup. Its name derives from the Latin word “quercetum” (after *quercus*, oak), from which it was first isolated [5] (Figure 3). The synthesis of this compound dates back to 1904 [11] and is associated with Polish chemist Kostanecki, who synthesized some flavonoids in 1898–1910 and thus went down in history as a pioneer of the chemistry of flavonoids [12]. In turn, Albert Szent Györgyi (Hungarian biochemist) was the first to discover dietary flavonols in 1936 [9]. One year later, he was awarded the Nobel Prize in Physiology or Medicine [6]. A few decades after these events (i.e., in the 1980s), studies on the antioxidative properties of flavonoids were started, and, in the early 1990s, the “French paradox”, described by Renaud and de Lorgeril in an article published in the Lancet journal in 1992, contributed to the renewal of interest in research on these phytonutrients (Figure 3).

Like V, QUE with its biological activity has aroused the interest of the global scientific community, and its medical effects are still extensively being studied. It has been found that this flavonol has many health benefits [13]. The high radical scavenging capacity and strong antioxidant properties of this bioactive compound [14] resulting from its chemical structure are frequently mentioned in the context of its pharmacological function. Studies on the capacity of QUE to scavenge some radicals showed a very high efficiency of this bioflavonoid (estimated at the level of 98% at 100 μM) in scavenging the 2,2-diphenyl-1-picrylhydrazyl radical (DPPH^●^). For comparison, the DPPH^●^ scavenging power of other flavonoids, i.e., morin, chrysin, and silibinin (at 100 μM) was estimated at 88%, 18%, and 16%, respectively [15]. Moreover, although it was markedly lower than in the case of DPPH^●^, the capacity of QUE to scavenge peroxyl radical (ROO^●^) was 26% higher than that of morin and silibinin, which were estimated at 5.2% and 5%, respectively [15]. Additionally, although the ability of QUE to scavenge the 2,2′-azino-bis(3-ethylbenzothiazoline-6-sulfonic acid (ABTS) radical cation is weak, it is still greater (4.7%) than that of morin (2%), chrysin (0.9%), and silibinin (1.8%) [15]. Thus, these findings clearly point to the high radical scavenging power of QUE. In addition, due to the presence of three chelating sites (i.e., 3′,4′-dihydroxyl, 3- or 5-hydroxyl, and 4-carbonyl groups), QUE is capable of forming complexes with many metal cations [16], thereby influencing metal bioavailability and serving as an antidote for metal poisoning. The complexes of QUE with metals are concisely summarized in the following section.

## 4. Metal-QUE Complexes—A Brief Outline

### 4.1. Complexation with Heavy Metals, Rare Earth Elements, and Elements with High Biological Importance

**Figure 4:**
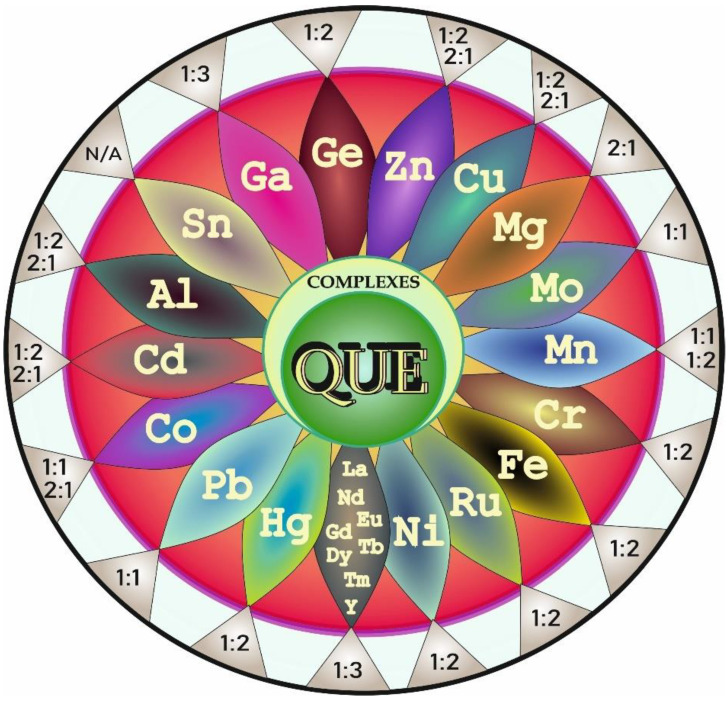
Metal-quercetin (QUE) complexes reported in the literature. N/A: not available.

According to the literature data, the increasing interest in studies on complexation of QUE with different elements dates back to the early 21st century. Since that time, many research groups worldwide have focused on the examination of this process. The summary of complexes formed between metals and QUE along with the metal (M):ligand (L = QUE) stoichiometric ratios is provided below (Figure 4).

It has been reported (Figure 4) that zinc (Zn) [17], copper (Cu) [18], magnesium (Mg) [19], iron (Fe) [20], ruthenium (Ru) [21], lead (Pb) [22], cobalt (Co) [23], cadmium (Cd) [23], gallium (Ga) [24], nickel (Ni) [25], aluminum (Al) [26], chromium (Cr) [27], manganese (Mn) [28], molybdenum (Mo) [29], mercury (Hg) [30], tin (Sn) [31], and germanium (Ge) [32] as well as rare earth metals such as lanthanum (La), neodymium (Nd), europium (Eu), gadolinium (Gd), terbium (Tb), dysprosium (Dy), thulium (Tm), and yttrium (Y) [33,34] are able to chelate with QUE to form QUE-metallic ion complexes. 

The literature review showed that the molecular structure, stability, and chelating sites of metal-QUE complexes as well as their antioxidant/cytotoxic properties and pharmacological effects have become the subjects of intensive experimental research. Special attention is given to the structure–activity relationships.

### 4.2. Variation in Antioxidant Activity 

Generally, complexes of flavonoids with metal ions can exhibit similar or novel characteristics, compared to the parent flavonoid [16]. Some studies of metal-QUE complexes have indicated that complexation of this polyphenolic phytochemical with metal ions may modify its chemical properties and affect, e.g., its antioxidant ability. The findings on the variations in the antioxidant activity of QUE after complexation with certain elements are collected in Table 1. 

The data provided above clearly show that metals such as Cr, Cu, Fe, Co, Cd, Mg, Ga, and Ru as well as rare earth elements (i.e., La, Nd, Eu, Gd, Dy, Tm, and Y) complexed with QUE improve the antioxidant activity of the compound, whereas Zn, Pb, Sn, and Tb, have an opposite effect, i.e., they reduce the antioxidative potential of this flavonol. As reported, hydrogen atom and/or electron donating mechanisms are linked to these effects.

### 4.3. Selected Events on the Timeline 

**Figure 5:**
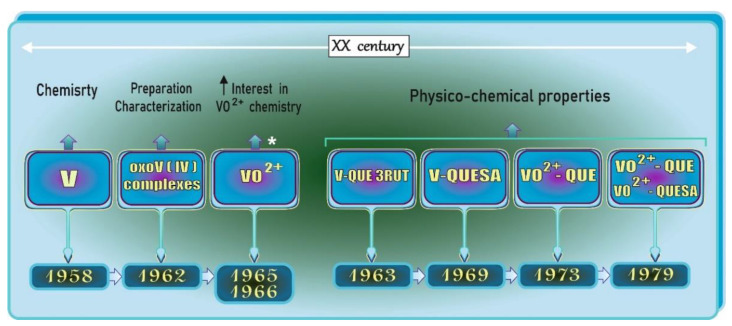
Selected events regarding vanadium chemistry and vanadium (V)-quercetin (QUE) complexes on the timeline. Elaborated on the basis of available literature data [7,8,36,37,38,39,40,41]. V: vanadium; QUE: quercetin; QUE 3RUT: QUE 3-rutinoside; QUESA: QUE-sulfonic acid; VO^2+^: oxovanadium cation (vanadyl). * after [42]. ↑: increase.

The aim of this section, which is a prelude to further parts, is to present the historical background of studies on V and QUE complexes and some events related to V chemistry. The description provided in this section is accompanied by Figure 5, in which selected points are presented in chronological order for the reader’s convenience.

**Figure 6:**
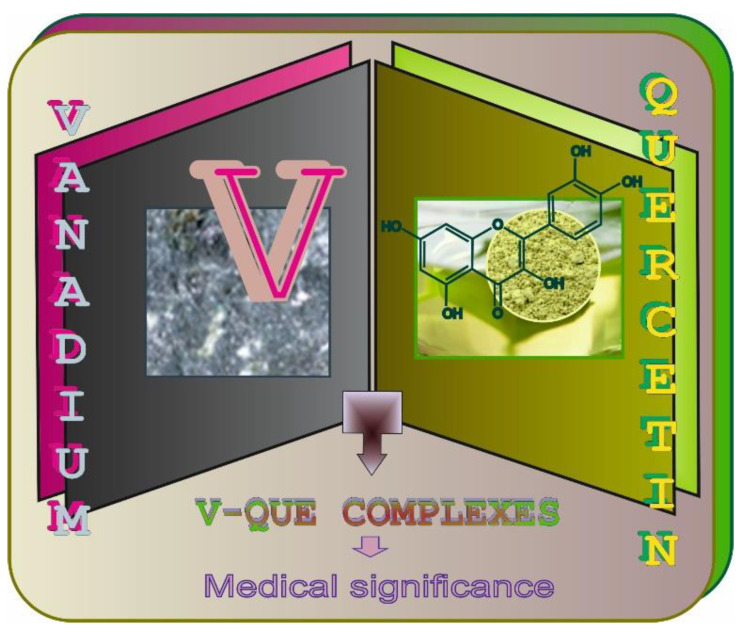
Vanadium-quercetin (V-QUE) complexes—the core issues of the present report.

Not long after André Morette comprehensively reviewed the V chemistry in 1958 (Figure 5), devoting 275 pages to this metal in a book titled *Nouveau Traité de Chimie Minérale* edited by Paul Pascal [36], and when two review articles focused on oxovanadium(IV) complexes were published in 1965 and 1966 [38,42], interest in the chemistry of oxovanadium(IV) species (VO^2+^) significantly increased [42]. Since that time, researchers have been working on the preparation and characterization of a number of new complexes of the oxovanadium(IV) cation [37] (Figure 5). For example, in 1963, Dev and Jain [7] found that V forms an orange water-soluble complex with QUE 3-rutinoside (QUE 3RUT) in the molar ratio of 1:2, respectively, and noted that QUE 3RUT can be employed for the spectrophotometric determination of this metal. A few years later, i.e., in 1969, another research group [8] reported that V forms a stable yellow complex with sulfonic derivatives of QUE, i.e., QUE-sulfonic acid (QUESA), in the molar ratio of 1:1. Other studies on the physico-chemical properties of the complexes between VO^2+^ and QUE or QUESA were performed in the following years, i.e., in 1973 and 1979 [39,40,41]. As emphasized by some authors, sulfonic derivatives of QUE, such as quercetin-5′-sulfonic acid, which is non-toxic and can form complex compounds with metals, may serve as an analytical reagent for spectrophotometric determination of elements [43] or as an antidote against their toxicity [44]. More details about V-QUE complexes in the context of their therapeutic potential, which are the core issues of the present paper (Figure 6), are collected and concisely summarized in later parts of this review. 

## 5. Biomedical Activity of V-QUE Complexes—Promising Therapeutic Effects

It should be highlighted that V is at the forefront among the different metals analyzed for their potential therapeutic use. This is mainly due to the anti-diabetic [45] and anti-tumoral [46] properties of this element. QUE also exerts anti-cancer activity [47] and has a positive effect on diabetes [48]. 

Both diabetes and cancer affect many people. The former is a life-long illness, whereas the latter (whose treatment is complex and often involves many modalities) is not always a one-time event. Both illnesses exert an impact on the quality of life and are accompanied by oxidative stress [49,50]. The two following sections summarize studies on both the anti-diabetic and anti-tumoral activities of V-QUE complexes.

### 5.1. Studies on Antidiabetic Potential—A Summarizing Note

**Figure 7:**
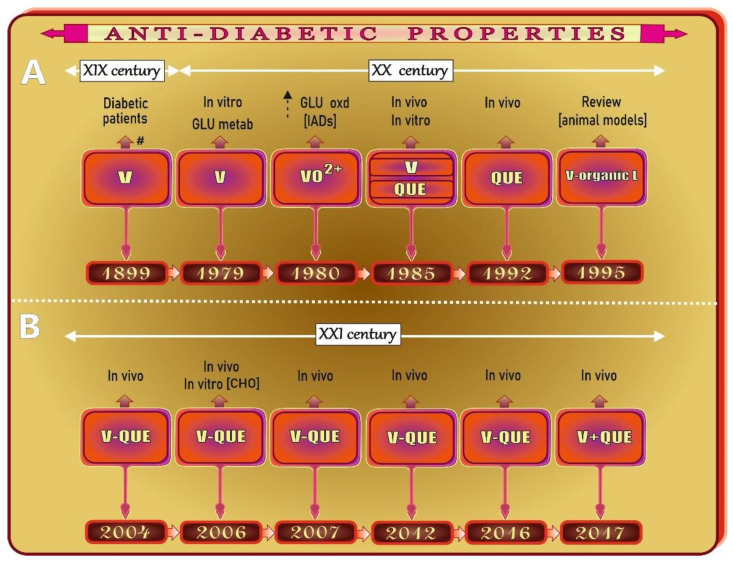
Summary of research on the vanadium (V)/quercetin (QUE) insulin-like effects (**A**) and potential antidiabetic activities of V-QUE complexes (**B**) on the timeline. Elaborated on the basis of available literature data [51,52,53,54,55,56,57,58,59,60,61,62,63]. CHO: Chinese hamster ovary cells; GLU: glucose; IADs: isolated adipocytes; L: ligand; Metab: metabolism; Oxd: oxidation; QUE: quercetin; V: vanadium; VO^2+^: oxovanadium cation (vanadyl). ^#^ after [51]. 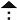 stimulation.

As shown in Figure 7, the first report on the role of V in hyperglycemia was published as early as in 1899, when reduced glycosuria was noted in diabetic patients after oral vanadate supplementation [51]. However, the results of one of the first studies exploring the effects of V compounds on various glucose (GLU) metabolic pathways were published much later. More precisely, in 1979, Tolman et al. [52] reported that both vanadate and vanadyl stimulate GLU oxidation and glycogen synthesis in vitro. In the same period, i.e., in 1980, Schechter and Karlish [53] showed insulin (INS)-like stimulation of GLU oxidation in rat adipocytes by vanadyl(IV) ions. After another five years, Heyliger and co-workers [54] revealed the INS-mimetic action of V in vivo in a rodent model for the first time. Interestingly, in the same year, Hii and Howell [55] demonstrated a stimulating effect of QUE on INS secretion in isolated rat islets of Langerhans. Seven years later, i.e., in 1992, Nuraliev and Avezov [56] noted the efficacy of QUE in diabetic rats (Figure 7), indicating that this phenolic compound is able to normalize the level of glycemia and elevate the hepatic glycogen content. In turn, in the mid-1990s (Figure 7), McNeill and co-workers published a review article titled “Increased potency of vanadium using organic ligand” and drew attention to organic V compounds that are effective INS-mimetic agents at lower doses, in comparison with inorganic V forms [57].

The first work on the hypoglycemic activity of one of the V-QUE complexes was published only at the beginning of the 21st century, in 2004 (Figure 7). The authors from India [58], who focused on the synthesis, structural properties, and INS-enhancing potential of bis(quercetinato)oxovanadium(IV) conjugate (BQOV), revealed highly potent INS-enhancing activity of BQOV in an animal model and concluded that this complex could be a valuable therapeutic agent used to treat type 1 and 2 diabetes. Two years later, the same research group [59] carried out in vitro (CHO cells) and in vivo (diabetic Balb/c mice) studies on the cytotoxicity and acute toxicity of BQOV (in which V was conjugated at the C3 and C4 positions of ring C in QUE) and vanadyl sulfate (VS) to check which of the two compounds is less toxic and more hypoglycemic. Reactive oxygen species (ROS) generation and antioxidant potential were examined as well. As reported, no significant alterations in ROS production were recorded in the kidney of diabetic animals treated with BQOV, compared to the untreated diabetic control group, while a 2-fold increase in the amount of ROS was noted after VS injection. In addition, the ROS production by BQOV in the CHO cells was negligible, compared to that in the VS-treated cells. Additionally, no statistically significant changes were demonstrated in the serum creatinine (Cre) and urea levels in the BQOV-treated mice, in comparison with the untreated diabetic ones, and no abnormalities were observed in the kidney of these animals in histopathological studies. In contrast, the administration of inorganic V salt (VS) resulted in a significant increase in both serum Cre and urea levels. Signs of acute tubular necrosis were recorded in this group of animals as well. Additionally, BQOV was demonstrated to normalize the blood GLU level more effectively than VS. Based on these data, it can be concluded that the toxicity of V is reduced after complexation with QUE. A year later (Figure 7), i.e., in 2007, the same research team conducted further studies on the effect of BQOV on carbohydrate metabolism and oxidative stress in diabetic animals [60]. They showed that 3-week oral administration of BQOV to diabetic mice led to a reduction in the blood GLU level, an increase in GLU uptake by the liver and skeletal muscle, and normalization of hepatic mRNA levels of glucose 6-phosphatase (G-6-Pase) and glucokinase (GK). The serum antioxidant capacity, which was significantly reduced in the diabetic BQOV-untreated animals, was restored to the control values by the BQOV treatment. The hepatic and pancreatic activity of some antioxidant enzymes/levels of malondialdehyde (MDA), which was elevated in the untreated diabetic animals, decreased in response to the BQOV administration. Moreover, the BQOV complex was shown not to cause any histopathological alterations in mouse liver and kidney. These findings clearly point to a reduction in oxidative stress generated in diabetes and to improvement of carbohydrate metabolism upon BQOV treatment. Another report on V-QUE complexes was published five years later, i.e., in 2012 (Figure 7). The investigations of the hypoglycemic and antilipidemic effects of a new V-QUE complex, i.e., di-*μ*-hydroxo-bis(quercetinatooxovanadium(IV) (HOBQOV), in which V was coordinated at the 4-carbonyl and 3-OH groups of QUE [61], showed that HOBQOV administered to diabetic rats for 15 days mitigated the elevated blood GLU level to nearly normal, and this compound turned out to be more effective than VS. It was also noted that the concentrations of triglycerides (TG), total cholesterol (T-CHOL), and the low-density lipoprotein fraction (LDL-CHOL) in the serum of the HOBQOV-supplied rats were lower than those in the untreated diabetic animals. In addition, in the case of T-CHOL and LDL-CHOL, the decrease was greater than that noted after the VS treatment. In turn, the serum level of the high-density lipoprotein fraction (HDL-CHOL) was higher in the HOBQOV-administered rats, compared to that in the untreated diabetic animals, and this increase was also greater than that observed after the VS treatment. To sum up, these results allow the conclusion that HOBQOV has a better influence on both glucidic and lipidic profiles in diabetic animals than VS. Two recent articles published in 2016 and 2017 (Figure 7) provided data on the effect of some organic V compounds, e.g., vanadyl-QUE (VS-QUE), on GLU homeostasis in diabetic mice [62] and on the influence of VS, QUE, and their mixture (VS+QUE) on glycemia and the lipid panel in diabetic rats [63]. The former study [62] showed that the VS-QUE conjugate administered orally to diabetic mice for 5 weeks markedly limited an increase in the level of GLU in the blood. In turn, the latter study [63] indicated that after induction of diabetes, the VS+QUE mixture more effectively reduced the blood GLU level than VS and QUE administered separately. Moreover, the VS+QUE mixture also reduced the T-CHOL level more effectively than QUE alone, whereas the VS treatment had no effect. Additionally, QUE administered separately and together with V elevated the HDL-CHOL concentration, but not significantly. Thus, the results of these two studies clearly showed the effectiveness of the V-QUE complex and V+QUE mixture in normalizing the glycemia level and lipid profile. The summary of the anti-diabetic properties of V, QUE, and V-QUE complexes along with other effects with respect to the data presented in Figure 7 is provided in Table 2.

### 5.2. Studies on Antitumoral Potential—A Summarizing Note

#### 5.2.1. First Studies on the Anticarcinogenic Effects of V, QUE, and V-QUE Complexes

**Figure 8:**
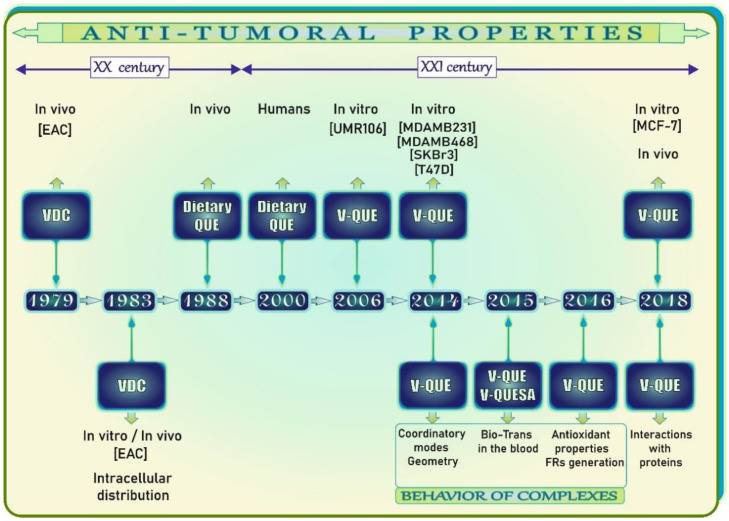
Summary of research on the vanadium (V)/quercetin (QUE) anticarcinogenic properties and antitumoral activities of V-QUE complexes as well as their behavior and interactions with proteins on the timeline. Elaborated on the basis of available literature data [64,65,66,67,68,69,70,71,72,73,74]. Bio-Trans: biotransformation; EAC: Ehrlich ascites carcinoma; FRs: free radicals; MCF-7: human breast cancer cell line; MDAMB231: human breast cancer cell line; MDAMB468: human breast cancer cell line; QUE: quercetin; SKBr3: human breast cancer cell line; T47D: human breast cancer cell line; UMR106: rat osteosarcoma cell line; VDC: vanadocene dichloride; V-QUE: vanadium-quercetin complex; V-QUESA: vanadium-quercetin sulfonic acid.

According to the literature data, the first paper on the antitumoral activity of V, published in the late 1970s (Figure 8), was focused on the antineoplastic properties of vanadocene dichloride (VDC) against Ehrlich ascites tumor in CF_1_ mice [64]. VDC was demonstrated to exhibit antineoplastic potential similar to that found for titanocene dichloride and cis-dichlorodiamine platinum(II). Four years later (Figure 8), Köpf-Maier and Krahl [65] analyzed the ultrastructural localization of V after in vitro and in vivo treatment of Ehrlich ascites tumor with VDC. They found that this metal mainly accumulated in the nuclear heterochromatin and, to a lesser extent, in the nucleolus and cytoplasmic ribosomes, which clearly pointed to nucleic acids as intracellular targets of V. In turn, in 1988 (Figure 8), Verma and co-workers [66] reported for the first time that dietary QUE inhibits 7,12-dimethylbenz[*a*]anthracene (DMBA)- and N-nitrosomethylurea (NMU)-induced mammary cancer in female rats and hypothesized that this flavonol may be an inhibitor of cancer induction in colon, lung, and intestine tissues.

At the beginning of the 21st century (Figure 8), Boyle and co-workers [67] conducted a study on the antioxidant effects of flavonoids, in which six healthy non-obese normocholesterolemic female volunteers (20–44 years of age) were fed with a flavonoid-rich meal, i.e., lightly fried onions (*Allium cepa*), a major QUE source [75], and with other low flavonoid foods and beverages. Individual concentrations of antioxidants and the total plasma antioxidant capacity as well as DNA damage and certain oxidative stress markers in the urine were determined in the study. The results showed that the plasma level of QUE-3-glucoside (QUE-3G) increased, whereas the DNA strand breakage decreased following the supplementation with the onion meal. Moreover, a significant decrease in the excretion of urinary 8-hydroxy-2′-deoxyguanosine (8OHdG), a well-known marker of oxidative DNA damage [76], was noted at 4 h following ingestion, and this decline corresponded with the maximal plasma QUE-3G level [67]. Thus, the results of this study provided tangible evidence for the protective effect of QUE-3G against DNA damage. 

In 2006–2018, findings from studies on the anti-tumoral properties and behavior of V-QUE complexes were published (Figure 8). One of the studies showed that the (VO(QUE)_2_EtOH)_n_ complex (1:2) administered in lower concentrations (2.5–20 µM) slightly stimulated the proliferation of a tumoral osteoblast cell line (UMR106), whereas inhibitory activity against proliferation of these cells was demonstrated at higher levels, i.e., 40–100 µM [68]. An inhibitory effect of QUE alone on the proliferation of UMR106 tumor cells was also found. Additionally, the osteogenic activity of the (VO(QUE)_2_EtOH)_n_ complex reflected in the stimulation of type I collagen production and a slight inhibitory effect on the activity of alkaline phosphatase (ALP) [68], which are the well-known markers of the osteoblast differentiation process [77], was noted. The authors suggested that VO(IV) was coordinated through the O carbonyl atom and the 3-OH or 5-OH groups of QUE. In 2014, the results of another study, carried out by Naso et al. [69] and aimed at exploration of the cytotoxic properties of the (VO(QUE)_2_EtOH)_n_ complex with regard to four human breast cancer cell lines, i.e., MDAMB231, MDAMB468, SKBr3, and T47D, showed that (VO(QUE)_2_EtOH)_n_ inhibited the viability in all breast cell lines at concentrations of 10 µM and 100 µM and stimulated the viability of normal breast epithelial cells at 10 µM. QUE was also found to be able to inhibit the viability in all of these tumor cell lines at both concentrations, and only a slight cytotoxic effect was observed in normal cells. The potent cytotoxic effect of the (VO(QUE)_2_EtOH)_n_ complex against the tested cell lines was reflected by the following IC_50_ values: 10 µM (MDAMB231), 23 µM (SKBr3), 7.4 µM (MDAMB468), and 5 µM (T47D). In turn, the IC_50_ values obtained for QUE, i.e., 50 µM (MDAMB231), 26 µM (SKBr3), 24 µM (MDAMB468), and 81.5 µM (T47D), were higher, which implies a lesser antitumoral potency of this flavonol, compared to that of the complex. Moreover, in the incubation with the MDAMB231 cells, the (VO(QUE)_2_EtOH)_n_ complex was also found to increase the activation of caspase 3/7, ROS production, and DNA damage (+34.2%, +29.6%, and +74%, respectively) [69]. As reported, V was complexed through the 4-carbonyl and the 3-OH groups of QUE. To the best of the author’s knowledge, another paper on the antitumor potential of V(IV)-QUE complex (1:2), in which V was coordinated at the 5-OH (ring A) and 4-carbonyl (ring C) groups of QUE, was published in 2018 [73]. It presents data from an in vitro study on human breast cancer cell line MCF-7 and from an in vivo tumorigenicity study in female Sprague–Dawley rats. As reported, the V(IV)-QUE complex protected against 7,12-dimethylbenz(α)anthracene (DMBA)-induced mammary carcinogenesis. A decrease in cell proliferation and an increase in the apoptotic index in the V(IV)-QUE treated rats, compared to the carcinogen control group (i.e., DMBA-challenged animals), was demonstrated. The mammary tissue in the V(IV)-QUE+DMBA-treated animals returned to nearly normal architecture. The V(IV)-QUE complex was also found to exhibit a dose- and time-dependent inhibitory effect on MCF-7 human mammary cancer cells. The authors recorded a reduction in cell viability to 83%, 71%, and 43.5% at concentrations of 125, 200, and 275 µM, respectively, compared to the control. They also noted the highest inhibition rate of 56% at the 275 µM concentration of the V(IV)-QUE complex at 48 h. The cycle arrest and induction of apoptosis by upregulation of p53, caspase 3/9, and Bax as well as downregulation of Bcl-2, VEGF, mTOR, and Akt were suggested as a mechanism by which the V(IV)-QUE complex acts as a chemotherapeutic agent in rat mammary carcinogenesis and the MCF-7 cell line. However, it should be emphasized that the in vitro doses used in the studies cited above were high, and they may not be achievable in vivo. Moreover, bearing in mind the fact that QUE metabolites may be responsible for the biological effects, basic pharmacokinetic work is necessary to explain the findings obtained from the in vivo studies more thoroughly. The summary of the anti-tumoral properties of V, QUE, and V-QUE complexes with respect to the data presented in Figure 8 is provided in Table 3.

#### 5.2.2. Antioxidant Capacity of V-QUE Complexes

The antioxidant properties and radical production capability of certain V^IV^O complexes formed by flavonoid ligands, e.g., QUE, were one of the subjects of researchers’ interest, and the bis-chelated species of QUE, i.e., (VO(QUE)_2_)^2^, was one of the compounds evaluated in this regard [72]. The data obtained from these studies indicated that the (VO(QUE)_2_)^2−^ complex has a higher antioxidant capacity than a complex with morin, and the amount of hydroxyl radicals (HO^●^) produced in Fenton-like reactions decreased when the 5,5-dimethyl-1-pyrroline *N*-oxide (DMPO) spin trapping assay of HO^●^ was performed in the system with (VO(QUE)_2_)^2−^. Moreover, it was also noted that in the absence of metal, i.e., in the QUE/H_2_O_2_/DMPO system, a small amount of the hydroxyl radical adduct (DMPO-OH) was formed, which indicated that QUE is able to reduce H_2_O_2_ to generate the HO^●^ radical. Morin, however, was not able to produce HO^●^ in the absence of the metal. These findings indicate that the specific structure of flavonoids and their ability to react with H_2_O_2_ are crucial in the modulation of ROS production, which may further be used in studies on their antitumoral efficacy. Another paper, published in 2018, provided data on the antioxidant potential of V(IV)-QUE measured with the ferric reducing antioxidant power (FRAP), 2,2′-azino-bis (3-ethylbenzothiazoline 6-sulphonate) (ABTS), and 2,2-diphenyl-1-picrylhydrazyl (DPPH) methods [73]. The following results: (a) FRAP value_10 min/40 mM compound_—1.5 (for V-QUE complex) and 1.2 (for QUE); ABTS radical scavenging activity—90.2% (for V-QUE complex) and 87.4% (for QUE); DPPH radical scavenging activity—~95% (for V-QUE complex) and ~86% (for QUE) showed that the free radical scavenging power of the V(IV)-QUE complex is slightly higher than that of QUE alone and provided grounds for concluding that the antioxidant activity of QUE is improved via complexation with V. For comparison, another research team [59] reported the FRAP value_4 min/100 mM compound_ for the BQOV complex and QUE of 4.88 and 5.43, respectively, and the superoxide dismutase (SOD) IC_50_ value of 0.63 mM and 0.58 mM for BQOV and QUE, respectively, which in turn shows that BQOV and QUE have comparable antioxidant capacity. Interestingly, the values of SOD IC_50_ and FRAP for V alone were 4.1 mM and nil, respectively [59]. Noteworthy also is the fact that the percentage of the DPPH^●^ radical scavenging power of such flavonoids as morin, naringenin, silibinin, and chrysin increases upon V^IV^O complexation [15]. The summary of data on the antioxidant properties of the V-QUE complex and free QUE is provided in Table 4.

#### 5.2.3. Studies on the Behavior of V-QUE Complexes—The Most Important Aspects

As stressed by some authors, recognition of the coordination modes and geometry of complexes between V and flavonoids will contribute to understanding their biotransformation in the blood and provide information on the active species in the organism [70]. A study on the coordination mode and geometry of potential antitumor oxidovanadium(IV) complexes formed by different flavonoids [70] showed that QUE forms anionic V^IV^O penta-coordinated complexes with square pyramidal geometry and (O^−^, O^−^) or “catechol-like” coordination. A year later, i.e., in 2015 (Figure 8), the results of studies on the biotransformation of V^IV^O complexes formed by QUE, i.e., VO-(QUE)_2_ and its sulfonic derivative, i.e., quercetin-5′-sulfonic acid (VO-(QUESA)_2_), in the plasma/erythrocytes revealed that both VO-(QUE)_2_ and VO-(QUESA)_2_ remain unchanged in the system with apo-transferrin and albumin and that VO-(QUE)_2_ and VO-(QUESA)_2_ cross the erythrocyte membrane and do not transform in the cytosol [71]. Three years later, Sciortino et al. [74] conducted a study using an integrative spectroscopic (electron paramagnetic resonance, EPR) and computational approach to elucidate the noncovalent interaction between bis-chelated V^IV^O-flavonoid complexes with anticancer activity and lysozyme (used to examine the metal complex–protein interaction). The results showed a gradual variation in the EPR spectra at ROOm temperature, which was linked to the strength of the interaction between the square pyramidal complexes and the surface lysozyme residues. It was noted that the strength of the interaction depended on the number of OH or CO groups of the ligands that can interact with different sites on the protein surface. The authors recorded a rigid limit (strong interaction) EPR spectrum for (VO(QUE)_2_)^2−^.

## 6. Biological Effects of V and QUE in a Nutshell

**Figure 9:**
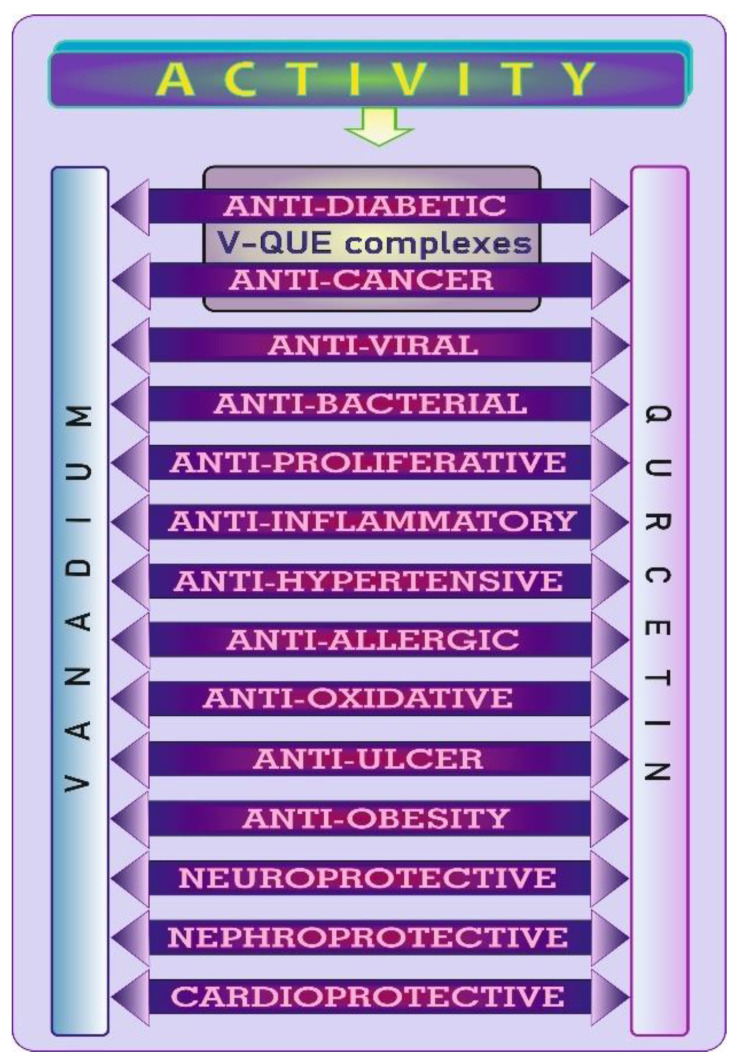
Biological properties of vanadium and quercetin.

The wide range of common biological activities of V and QUE are graphically summarized in Figure 9. V and QUE show anti-viral [78,79], anti-bacterial [3,80], anti-proliferative [81,82], anti-inflammatory [78,79,83], anti-hypertensive [3,84], anti-allergic [79,85], anti-oxidative [86,87], anti-cancer [88,89], anti-diabetic [3,90], anti-ulcer [91,92,93], and anti-obesity activity [3,94] as well as neuroprotective [79,95], nephroprotective [96,97], and cardioprotective effects [97,98]. Such a wide range of activities of V and QUE indicates their potential therapeutic benefits and provides a basis for further studies of this unique class of pharmacological agents.

## 7. Summary and Conclusions

The studies reviewed above clearly showed that V-QUE complexes have antidiabetic and antitumoral potential. They also revealed their antioxidant activity and radical scavenging power. These properties, supported by the experimental evidence collected in this review, point to the promising effects of V-QUE complexes in the treatment of diabetes and cancer and suggest that they can be developed as pharmacological agents for both illnesses. 

On the other hand, the analysis of available literature data also revealed that further studies on the metal-flavonoid complexes are necessary in the context of their potential as therapeutic drugs. Studies focused on structural modifications of QUE improving its bioavailability and identification of cellular targets of its complexes as well as detailed elucidation of the mechanisms of action and biotransformations of metallic complexes of this polyphenolic flavonoid will be critical for the development of effective therapeutic approaches in which these complexes could be used. Moreover, pharmacokinetic studies are also needed to determine not only their bioavailability in vivo but also to define the crucial metabolites of V-QUE complexes in the blood after supplementation. The knowledge in this research field would provide a solid basis for further research on the development of new drugs that could be exploited in medicine in the future.

## Figures and Tables

**Table 1:** Summary of findings on variations in the antioxidant potential of metal-QUE complexes vs. QUE—the role of metals.

Complexes	Antioxidant Ability *		Mechanism	References
	M-QUE CoL	QUE ^#^		
Cr-QUE	++	+	H transferring mechanism e^−^ donating mechanism (↑ efficiency of H-atom donation)	[27]
Cu-QUE	++	+		[18,35]
Fe-QUE	++	+		[20]
Co-QUE	++	+		[23]
Cd-QUE	++	+		[23]
Mg-QUE	++	+		[19]
Ga-QUE	++	+		[24]
Ru-QUE	++	+		[21]
REE-QUE	++	+		[33]
Zn-QUE	+	++	↓ e^−^ transfer from QUE by M chelation (↓ e^−^ donating ability in the complex)	[35]
Pb-QUE	+	++		[22]
Sn-QUE	+	++		[31]
Tb-QUE	+	++		[34]

M: metal; QUE: quercetin; CoL: complex; e^−^: electron; H: hydrogen; Cr: chromium; Co: copper; Fe: iron; Co: cobalt; Cd: cadmium; Ga: gallium; Ru: ruthenium; Zn: zinc; Pb: lead; Sn: tin; Tb: terbium; REE: rare earth elements = La: lanthanum; Nd: neodymium; Eu: europium; Gd: gadolinium; Dy: dysprosium; Tm: thulium; Y: yttrium. * Expressed as ++/+: greater/lower. ^#^ Free quercetin. ↑: increase; ↓: decrease.

**Table 2:** Summary of anti-diabetic properties of vanadium, quercetin, and vanadium-quercetin complexes along with other effects: human studies and in vivo/in vitro models.

Humans/ Animals/Cells/Tissues	Compound	Treatment	Effects	References
Humans				
Diabetic patients	Na_3_VO_4_	oral supplementation	↓ glycosuria	[51]
In vivo model				
Diabetic Wistar rats ( 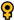 )	Na_3_VO_4_	0.6 to 0.8 mg/mL *per os*, 4 wk	↓ GLU_P_ level	[54]
Diabetic rats	QUE	10 and 50 mg/kg	↑ Gly_L_ content Normalization of glycemia	[56]
Diabetic Balb/c mice ( 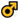 )	BQOV	at a one-time dose of 0.4 mmol/kg BW *per os*	↓ GLU_B_ level	[58]
Diabetic Balb/c mice	BQOV	0.1 mmol V/kg BW i.p. up to 24 h	↓↓ GLU_B_ level → ROS_K_ production → Cre_S_ level, → urea_S_ level No histopathol. alterations in K	[59]
Diabetic Balb/c mice	VS	0.1 mmol V/kg BW i.p. up to 24 h	↓ GLU_B_ level ↑ ROS_K_ production ↑ Cre_S_ and urea_S_ levels Signs of ATN in K	[59]
Diabetic Balb/c mice ( 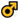 )	BQOV	0.2 mmol/kg *per os*, 3 wk	↓ GLU_B_ level ↑ GLU uptake by L and SM Normalization of mRNA levels of G-6-Pase_L_ and GK_L_ Normalization of some AE activities in L and PC ↓ MDA_L_/↓ MDA_PC_ levels No histopathol. alterations in L/K	[60]
Diabetic Wistar rats ( 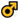 )	HOBQOV	0.4 mmol/kg BW/d *per os*, 15 d	Normalization of GLU_B_ level ↓ TG_S_, T-CHOL_S_, LDL-CHOL_S_ levels ↑ HDL-CHOL_S_ level	[61]
Diabetic K mice ( 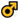 )	VS-QUE	0.1 mL/10 g BW at a dose of 80 mg/kg BW *per os*, 5 wk	Normalization of GLU_B_ level	[62]
Diabetic Wistar rats ( 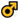 )	VS+QUE	0.01 mmol/kg BW+ 0.02 mmol/kg BW *per os*, 4 wk	Normalization of GLU_B_ level ↓ T-CHOL_S_, 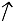 HDL-CHOL_S_ levels	[63]
In vitro model				
IADS	VS/NaVO_3_	0.6 mM/0.8 mM	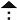 GLU oxidation	[52]
Isolated rat diaphragm	VS/NaVO_3_ Na_3_VO_4_/NH_4_VO_3_	0.5 mM/0.8 mM 0.54 mM/0.85 mM	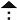 GLU conversion to Gly	[52]
Isolated rat hepatocytes	VS/NaVO_3_ Na_3_VO_4_/NH_4_VO_3_	0.25 mM/0.4 mM 0.27 mM/0.42 mM	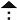 Gly synthesis	[52]
IADS	Na_3_VO_4_	0.1 mM	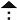 GLU oxidation	[53]
IOL	QUE	0.01–0.1 mmol/L	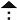 INS secretion	[55]

K mice: Kunming mice; V: vanadium; QUE: quercetin; V-QUE COP: vanadium-quercetin complex; NaVO_3_: sodium metavanadate; Na_3_VO_4_: sodium orthovanadate; NH_4_VO_3_: ammonium metavanadate; BQOV: bis(quercetinato)oxovanadium(IV) conjugate; VS: vanadium sulfate; HOBQOV: di-*μ*-hydroxo-bis(quercetinatooxovanadium(IV)); VS-QUE: vanadyl-quercetin conjugate; VS+QUE: vanadyl + quercetin mixture; ATN: acute tubular necrosis; GLU: glucose; Gly: glycogen; AE: antioxidant enzymes; IADS: isolated adipocytes; IOL: islets of Langerhans; INS: insulin; SM: skeletal muscle; ROS: reactive oxygen species; Cre: creatinine; G-6-Pase: glucose 6-phosphatase; GK: glucokinase; MDA: malondialdehyde; TG: triglyceride; T-CHOL: total cholesterol; LDL-CHOL: low-density lipoprotein fraction; HDL-CHOL: high-density lipoprotein fraction; d: days; wk: weeks; BW: body weight; B: blood; P: plasma; S: serum, L: liver; K: kidney; PC: pancreas. ↓: decrease; ↓↓: enhanced reduction; ↑: increase; →: without changes; i.p.: intraperitoneally. 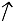: trend toward an increase; 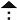: stimulation.

**Table 3:** Summary of anti-tumoral properties of vanadium, quercetin, and vanadium-quercetin complexes along with other effects with respect to data presented in Figure 8: human studies and in vivo/in vitro models.

Humans/ Animals/Cells	Compound/Diet	Treatment	Effects	References
Humans				
Healthy volunteers ( 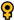 , 22–44 yr, *n* = 6)	FLAV-rich meal (QUE source)	oral supplementation 200 g LFO + other low FLAV F&B	↑ QUE-3G_P_ level ↓ DNA strand breakage ↓ 8OHdG_U_ level	[67]
In vivo model				
Carcinogen challenged CF_1_ mice	VDC	80 or 90 mg/kg 24 h after transplantation	100% tumor inhibition until d 30	[64]
Carcinogen ^‡^ challenged S-P rats ( 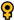 )	QUE	2% and 5% QUE diet 1 wk before carcinogen administration	↓ incidence/↓ number of MC	[66]
Carcinogen ^†^ challenged S-P rats ( 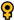 )	V(IV)-QUE	20 mg/kg BW *per os*, 24 wk 45 mg/kg BW *per os*, 24 wk	↓ proliferation ↑ AI, ↑ p53, ↑ Bax, ↓ Bcl2 ↓ proliferation ↑ AI, ↑ p53, ↑ Bax, ↓ Bcl2	[73]
In vitro model				
UMR106	[VO(QUE)_2_EtOH]_n_ QUE	40–100 μM, 24 h 20–100 μM, 24 h	↓ proliferation ↓ proliferation	[68]
MDAMB231	[VO(QUE)_2_EtOH]_n_	10 μM and 100 μM, 48 h	↓ viability	[69]
MDAMB468	[VO(QUE)_2_EtOH]_n_	10 μM and 100 μM, 48 h	↓ viability	
SKBr3	[VO(QUE)_2_EtOH]_n_	10 μM and 100 μM, 48 h	↓ viability	
T47D	[VO(QUE)_2_EtOH]_n_	10 μM and 100 μM, 48 h	↓ viability	
MDAMB231	QUE	10 μM and 100 μM, 48 h	↓ viability	
MDAMB468	QUE	10 μM and 100 μM, 48 h	↓ viability	
SKBr3	QUE	10 μM and 100 μM, 48 h	↓ viability	
T47D	QUE	10 μM and 100 μM, 48 h	↓ viability	
MDAMB231	[VO(QUE)_2_EtOH]_n_	25 μM, 24 h	↑ CASP 3/7 ↑ ROS ↑ DNA damage	[69]
MCF-7	V(IV)-QUE	125 μM, 48 h 200 μM, 48 h 275 μM, 48 h	↓ viability ↓ viability ↓ viability	[73]

S-P rats: Sprague–Dawley rats; FLAV: flavonoids; LFO: lightly fried onion; FLAV F&B: flavonoid foods and beverages; QUE-3G: quercetin-3-glucoside; 8OHdG: 8-hydroxy-2′deoxyguanosine; VDC: vanadocene dichloride; QUE: quercetin; UMR106: tumoral osteoblast cell line; MDAMB231: human breast cancer cell line; MDAMB468: human breast cancer cell line; SKBr3: human breast cancer cell line; T47D: human breast cancer cell line; MCF-7: human breast cancer cell line; CASP: caspase; ROS: reactive oxygen species; AI: apoptotic index; MC: mammary cancer; DMBA: 7,12-dimethylbenz(*a*)anthracene; NMU: N-nitrosomethylurea; h: hour; d: day; wk: week; yr: years; P: plasma; U: urine. ^‡^ DMBA or NMU; ^†^ : DMBA only. ↓: decrease; ↑: increase.

**Table 4:** Summary of results for antioxidant properties of the V-QUE complex and free QUE evaluated with different methods.

Methods	Antioxidant Activity				Reference
	V-QUE Complex		QUE		
DPPH (%)	95		86		[73]
ABTS (%)	90.2		87.4		
FRAP	1.5		1.2		
FRAP	4.88		5.43		[59]
SOD IC_50_	0.63		0.58		
DPPH (%)	ND		98		[15]
SOD IC_50_	ND		1.6		
ABTS (%)	ND		4.7		
ROO^●^	ND		26.1		
	QUE (free)	QUE (complex)		(VO (QUE)_2_)^2^	[72]
DPPH EC_50_ (UV-Vis)	5.3 × 10^−6^ M	4.7 × 10^−6^ M		2.4 × 10^−6^ M	
DPPH EC_50_ (EPR)	4.3 × 10^−6^ M	4.2 × 10^−6^ M		2.1 × 10^−6^ M	
	QUE versus V^IV^O^2+^		V^IV^O^2+^/QUE versus V^IV^O^2+^		
DMPO-OH (%)	4.2 vs. 100		30.9 vs. 100		

V: vanadium; QUE: quercetin; DPPH: 2,2-diphenyl-1-picrylhydrazyl; ABTS: 2,2′-azino-bis 3-ethylbenzothiazoline 6-sulphonic acid; FRAP: ferric reducing antioxidant power; SOD: superoxide dismutase; ROO^●^: peroxyl radical; V^IV^O^2+^: oxovanadium cation; DMPO: 5,5-dimethyl-1-pyrroline *N*-oxide; DMPO-OH: hydroxyl radical adduct; ND: not determined.

## References

[1] Rheder D. (1991). The bioinorganic chemistry of vanadium. Angew. Chem. Int. Engl..

[2] Richards R.L. (2006). Vanadium: Inorganic and Coordination Chemistry. Encyclopedia of Inorganic Chemistry.

[3] Ścibior A., Pietrzyk Ł., Plewa Z., Skiba A. (2020). Vanadium: Risks and possible benefits in the light of a comprehensive overview of its pharmacotoxicological mechanisms and multi-applications with a summary of further research trends. J. Trace Elem. Med. Biol..

[4] The Editors of Encyclopaedia Britannica “vanadium”. Encyclopedia Britannica, 18 June 2021. https://www.britannica.com/science/vanadium.

[5] Quercetin. Merriam-Webster.com Dictionary, Merriam-Webster. https://www.merriam-webster.com/dictionary/quercetin.

[6] MLA Style: The Nobel Prize in Physiology or Medicine 1937. NobelPrize.org. Nobel Prize Outreach AB 2022. Mon. 31 January 2022. https://www.nobelprize.org/prizes/medicine/1937/summary/.

[7] Dev B., Jain B.D. (1963). Spectrophotometric Determination of Vanadium(V) with Rutin (Quercetin 3-Rutinoside). Proc. Indian Acad. Sci. Sect. A.

[8] Yonekubo T., Satake M., Matsumoto T. (1969). Spectrophotometric determination of vanadium (V) with quercetin-sulfonic acid. Bunseki Kagaku.

[9] Bioflavonoids, Encyclopedia.com. Updated 18 May 2018. https://www.encyclopedia.com/medicine/diseases-and-conditions/pathology/bioflavonoids.

[10] Renaud S., De Lorgeril M. (1992). Wine, alcohol, platelets, and the French paradox for coronary heart disease. Lancet.

[11] Mitchell L.J. (1965). Survey of Previous Work of Quercetin. In Spectrophotometry of Molybdenum, Tungsten, and Chromium Chelates of Quercetin. Ph.D. Thesis.

[12] Chemistry International—Newsmagazine for IUPAC (1998). Polish chemistry. Chem. Int. Newsmag. IUPAC.

[13] Kim J.K., Park S.U. (2018). Quercetin and its role in biological functions: An updated review. EXCLI J..

[14] Bentz A.B. (2017). A Review of Quercetin: Chemistry, antioxident properties, and bioavailability. JYI.

[15] Islas M.S., Naso L.G., Lezama L., Valcarcel M., Salado C., Roura-Ferrer M., Ferrer E.G., Williams P.A.M. (2015). Insights into the mechanisms underlying the antitumor actovoty of an oxidovanadium(IV) compound with the antioxidant naringenin. Albumin binding studies. J. Inorg. Chem..

[16] Samsonowicz M., Regulska E. (2017). Spectroscopic study of molecular structure, antioxidant activity and biological effects of metal hydroxyflavonol complexes. Spectrochim. Acta Part A.

[17] Tan J., Wang B., Zhu L. (2009). DNA binding, cytotoxicity, apoptotic inducing activity, and molecular modeling study of quercetin zinc(II) complex. Bioorg. Med. Chem..

[18] Bukhari S.B., Memon S., MahROOf-Tahir M., Bhanger M.I. (2009). Synthesis, characterization and antioxidant activity copper–quercetin complex. Spectrochim. Acta A Mol. Biomol. Spectrosc..

[19] Ghosh N., Chakraborty T., Mallick S., Mana S., Singha D., Ghosh B., Roy S. (2015). Synthesis, characterization and study of antioxidant activity of quercetin-magnesium complex. Spectrochim. Acta A Mol. Biomol. Spectrosc..

[20] Raza A., Xu X., Xia L., Xia C., Tang J., Ouyang Z. (2016). Quercetin-iron complex: Synthesis, characterization, antioxidant, DNA binding, DNA cleavage, and antibacterial activity studies. J. Fluoresc..

[21] Roy S., Das R., Ghosh B., Chakraborty T. (2018). Deciphering the biochemical and molecular mechanism underlying the in vitro and in vivo chemotherapeutic efficacy of ruthenium quercetin complex in colon cancer. Mol. Carcinog..

[22] Ravichandran R., Rajendran M., Devapiriam D. (2014). Structural characterization and physicochemical properties of quercetin—Pb complex. J. Coord. Chem..

[23] Trifunschi S., Ardelean D. (2016). Synthesis, characterization and antioxidant activity of Co (II) and Cd (II) complexes with quercetin. Rev. Chim..

[24] Do Nascimento Simões V., Favarin L., Cabeza N., De Oliveira T., Fiorucci A., Stropa J., Rodrigues D., Cavalheiro A., Dos Anjos A. (2013). Synthesis, characterization and study of the properties of a new mononuclear quercetin complex containing Ga(III) ions. Química Nova.

[25] Tan J., Zhu L., Wang B. (2009). DNA binding and cleavage activity of quercetin nickel(II) complex. Dalton Trans..

[26] Cornard J.P., Merlin J.C. (2002). Spectroscopic and structural study of complexes of quercetin with Al(III). J. Inorg. Biochem..

[27] Chen W., Sun S., Cao W., Liang Y., Song J. (2009). Antioxidant property of quercetin–Cr(III) complex: The role of Cr(III) ion. J. Mol. Struct..

[28] Jun T., Bochu W., Liancai Z. (2007). Hydrolytic cleavage of DNA by quercetin manganese(II) complexes. Colloids Surf. B Biointerfaces.

[29] Ahmadi S.M., Dehghan G., Hosseinpourfeizi M.A., Dolatabadi J.E., Kashanian S. (2011). Preparation, characterization, and DNA binding studies of water-soluble quercetin--molybdenum(VI) complex. DNA Cell Biol..

[30] Bravo A., Anacona J.R. (2001). Metal complexes of the flavonoid quercetin: Antibacterial properties. Transit. Met. Chem..

[31] Dehghan G., Khoshkam Z. (2012). Tin(II)-quercetin complex: Synthesis, spectra characterisation and antioxidant activity. Food Chem..

[32] Zhai G., Zhu W., Duan Y., Qu W., Yan Z. (2012). Synthesis, characterization and antitumor activity of the germanium-quercetin complex. Main Group Met. Chem..

[33] Zhou J., Wang L., Wang J., Tang N. (2001). Synthesis, characterization, antioxidative and antitumor activities of solid quercetin rare earth(III) complexes. J. Inorg. Biochem..

[34] Ezzati Nazhad Dolatabadi J., Mokhtarzadeh A., Ghareghoran S.M., Dehghan G. (2014). Synthesis, characterization and antioxidant property of quercetin-Tb(III) complex. Adv. Pharm. Bull..

[35] Bratu M.M., Birghila S., Miresan H., Negreanu-Pirjol T., Prajitura C., Calinescu M. (2014). Biological activities of Zn(II) and Cu(II) complexes qith quercetin and rutin: Antioxidant properties and UV-protection capacity. Rev. Chim..

[36] Morette A., Foex M., Rohmer R., Haïssinsky M., Bouissières G., Pascal P. (1958). Vanadium-niobium-tantale-protactinium. Nouveau Traité de Chimie Minérale.

[37] Selbin J., Holmes L.H. (1962). Complexes of oxovanadium(IV). J. Inorg. Nucl. Chem..

[38] Selbin J. (1965). The chemistry of oxovanadium(IV). Chem. Rev..

[39] Chernaya N.V., Matyasho V.G. (1973). Vanadium(IV) quercetin complex in methanol and water-methanol solutions. Ukr. Khim. Zh..

[40] Kaushal G.P., Sekhon B.S., Bhatia I.S. (1979). Spectrophotometric determination of quercetin with VO^2+^. Mikrochim. Acta.

[41] Kopach M., Novak D. (1979). Vanadium(IV) complexes with quercitin-5’-sulfonic acid. Zh. Neorg. Khim..

[42] Selbin J. (1966). Oxovanadium(IV) complexes. Coord. Chem. Rev..

[43] Kopacz M., Bujonek B., Nowak D., Kopacz S. (2001). Studies of the equlibria of complexes of Pr(III), Nd(III), Eu(III), Gd(III), Dy(III) and Er(III) with quercetin-5’-sulfonic acid in aqueous solutions. Chem. Anal..

[44] Kopacz M., Kuźniar A. (2003). Complexes of cadmium (II), mercury (II) and lead (II) with quercetin-5’-sulfonic acid (QSA). Pol. J. Chem..

[45] Srivastava A.K., Mehdi M.Z. (2005). Insulino-mimetic and anti-diabetic effects of vanadium compounds. Diabet. Med..

[46] Crans D.C., Yang L., Haase A., Yang X., Sigel A., Sigel. H., Freisinger E., Sigel R.K.O. (2018). Health benefits of vanadium and its potential as an anticancer agent. Metallo-Drugs: Development and Action of Anticancer Agents, Metal Ions in Life Science.

[47] Gibellini L., Pinti M., Nasi M., Montagna J.P., De Biasi S., Roat E., Bertoncelli L., Cooper E.L., Cossarizza A. (2011). Quercetin and cancer chemoprevention. Evid. Based Complem. Altern. Med..

[48] Bule M., Abdurahman A., Nikfar S., Abdollahi M., Amini M. (2019). Antidiabetic effect of quercetin: A systematic review and meta-analysis of animal studies. Food Chem. Toxicol..

[49] Matough F.A., Budin S.B., Hamid Z.A., Alwahaibi N., Mohamed J. (2012). The role of oxidative stress and antioxidants in diabetic complications. Sultan Qaboos Univ. Med. J..

[50] Zhu B., Qu S. (2022). The relationship between diabetes mellitus and cancers and its underlying mechanisms. Front. Endocrinol..

[51] Gruzewska K., Michno A., Pawelczyk T., Bielarczyk H. (2014). Essentiality and toxicity of vanadium supplements in health and pathology. J. Physiol. Pharmacol..

[52] Tolman E.L., Barris E., Burns M., Pansini A., Partridge R. (1979). Effects of vanadium on glucose metabolism in vitro. Life Sci..

[53] Shechter Y., Karlish S.J.D. (1980). Insulin-like stimulation of glucose oxidation in rat adipocytes by vanadyl (IV) ions. Nature.

[54] Heyliger C.E., Tahiliani A.G., McNeill J.H. (1985). Effect of vanadate on elevayed blood glucose and depressed cardiac performance of diabetic rats. Science.

[55] Hii C.S., Howell S.L. (1985). Effects of flavonoids on insulin secretion and ^45^Ca^2+^ handling in rat islets of Langerhans. J. Endocr..

[56] Nuraliev I.N., Avezov G.A. (1992). The efficacy of quercetin in alloxan diabetes. Eksp. Klin. Farmakol..

[57] McNeill J.H., Yuen V.G., Dai S., Orvig C. (1995). Increased potency of vanadium using organic ligands. Mol. Cell Biochem..

[58] Shukla R., Barve V., Padhye S., Bhonde R. (2004). Synthesis, structural properties and insulin-enhancing potential of bis(quercetinato)oxovanadium(IV) conjugate. Bioorg. Med. Chem. Lett..

[59] Shukla R., Barve V., Padhye S., Bhonde R. (2006). Reduction of oxidative stress induced vanadium toxicity by complexing with a flavonoid, quercetin: A pragmatic therapeutic approach for diabetes. Biometals.

[60] Shukla R., Padhye S., Modak M., Ghaskadbi S.S., Bhonde R.R. (2007). Bis(quercetinato)oxovanadium IV Reverses Metabolic Changes in Streptozotocin-Induced Diabetic Mice. Rev. Diabet. Stud..

[61] Velescu B.S., Uivarosi V., Negres S. (2012). Effect of di-μ-hydroxo-bis (quercetinatooxovanadium(IV)) complex on alloxan-induced diabetic rats. Farmacia.

[62] Jiang P., Dong Z., Ma B., Ni Z., Duan H., Li X., Wang B., Ma X., Wei Q., Ji X. (2016). Effect of vanadyl rosiglitazone, a new insulin-mimetic vanadium complexes, on glucose homeostasis of diabetic mice. Appl. Biochem. Biotechnol..

[63] Velescu B.S., Anuta V., Aldea A., Jinga A., Cobeleschi P.C., Zbârcea C.E., Uivarosi V. (2017). Evaluation of protective effects of quercetin and vanadyl sulphate in alloxan induced diabetes model. Farmacia.

[64] Köpf-Maier P., Köpf H. (1979). Vanadocen-dichlorid–ein weiteres antitumor-agens aus der metallocenreihe. Z. Nat..

[65] Köpf-Maier P., Krahl D. (1983). Tumor inhibition by metallocenes: Ultrastructiral localization of titanium and vanadium in treated tumor cells by electron energy loss spectroscopy. Chem. Biol. Interact..

[66] Verma A.K., Johnson J.A., Gould M.N., Tanner M.A. (1988). Inhibition of 7,12-dimethylbenz(*a*)anthracene- and *N*-nitrosomethylurea-induced rat mammary cancer by dietary flavonol quercetin. Cancer Res..

[67] Boyle S.P., Dobson V.L., Duthie S.J., Kyle J.A.M., Collins A.R. (2000). Absorption and DNA protective effects of flavonoid glycosides from an onion meal. Eur. J. Nutr..

[68] Ferrer E.G., Salinas M.V., Correa M.J., Naso L., Barrio D.A., Etcheverry S.B., Lezama L., Rojo T., Williams P.A. (2006). Synthesis, characterization, antitumoral and osteogenic activities of quercetin vanadyl(IV) complexes. J. Biol. Inorg. Chem..

[69] Naso L., Valcarcel M., Villacé P., Roura-Ferrer M., Salado C., Ferrer E.G., Williams P.A.M. (2014). Specific antitumor activities of natural and oxovanadium(IV) complexed flavonoids in human breast cancer cells. New, J. Chem..

[70] Sanna D., Ugone V., Lubinu G., Micera G., Garribba E. (2014). Behavior of the potential antitumor V^IV^O complexes formed by flavonoid ligands. 1. Coordination modes and geometry in solution and at the physiological pH. J. Inorg. Biochem..

[71] Sanna D., Ugone V., Pisano L., Serra M., Micera G., Garribba E. (2015). Behavior of the potential antitumor V^IV^O complexes formed by flavonoid ligands. 2. Characterization of sulfonate derivatives of quercetin and morin, interaction with the bioligands of the plasma and preliminary biotransformation studies. J. Inorg. Biochem..

[72] Sanna D., Ugone V., Fadda A., Micera G., Garribba E. (2016). Behavior of the potential antitumor V^IV^O complexes formed by flavonoid ligands. 2. Antioxidant properties and radical production capability. J. Inorg. Biochem..

[73] Roy S., Banerjee S., Chakraboborty T. (2018). Vanadium quercetin complex attenuates mammary cancer by regulating the P53, Akt/mTOR pathway and downregulates cellular proliferation correlated with increased apoptotic events. Biometals.

[74] Sciortino G., Sanna D., Ugone V., Lledós A., Maréchal J.-D., Garribba E. (2018). Decoding Surface interaction of V^IV^O metallodrug candidates with lysozyme. Inorg. Chem..

[75] Iqbal M.S., Iqbal Z., Ansari M.I. (2019). Enhancement of total antioxidants and favonoid (quercetin) by methyl jasmonate elicitation in tissue cultures of onion (*Allium cepa* L.). Acta Agrobot..

[76] Valavanidis A., Vlachogianni T., Fiotakis C. (2009). 8-hydroxy-2′-deoxyguanosine (8-OHdG): A critical biomarker of oxidative stress and carcinogenesis. J. Environ. Sci. Health Part C.

[77] Huang W., Yang S., Shao J., Li Y.P. (2007). Signaling and transcriptional regulation in osteoblast commitment and differentiation. Front. Biosci..

[78] Semiz S. (2022). Vanadium as potential therapeutic agent for COVID-19: A Focus on its antiviral, antiinflamatory, and antihyperglycemic effects. J. Trace Elem. Med. Biol..

[79] Anand David A.V., Arulmoli R., Parasuraman S. (2016). Overviews of biological importance of quercetin: A bioactive flavonoid. Pharmacogn. Rev..

[80] Wang S., Yao J., Zhou B., Yang J., Chaudry M.T., Wang M., Xiao F., Li Y., Yin W. (2018). Bacteriostatic effect of quercetin as an antibiotic alternative in vivo and its antibacterial mechanism in vitro. J. Food Prot..

[81] Pisano M., Arru C., Serra M., Galleri G., Sanna D., Garribba E., Palmieri G., Rozzo C. (2019). Antiproliferative activity of vanadium compounds: Effects on the major malignant melanoma molecular pathways. Metallomics.

[82] Zhaorigetu, Farrag I.M., Belal A., Badawi M.H.A., Abdelhady A.A., Galala F.M.A.A., El-Sharkawy A., El-Dahshan A.A., Mehany A.B.M. (2021). Antiproliferative, apoptotic effects and suppression of oxidative stress of quercetin against induced toxicity in lung cancer cells of rats: In vitro and in vivo study. J. Cancer.

[83] Tripathi D., Mani V., Pal R.P. (2018). Vanadium in biosphere and its role in biological processes. Biol. Trace Elem. Res..

[84] Perez-Vizcaino F., Duarte J., Jimenez R., Santos-Buelga C., Osuna A. (2009). Antihypertensive effects of the flavonoid quercetin. Pharmacol. Rep..

[85] Li X., Lu Y., Yang J.H., Jin Y., Hwang S.L., Chang H.W. (2012). Natural vanadium-containing Jeju groundwater inhibits immunoglobulin E-mediated anaphylactic reaction and suppresses eicosanoid generation and degranulation in bone marrow derived-mast cells. Biol. Pharm. Bull..

[86] Matsubara T., Musat-Marcu S., Misra H.P., Dhalla N.S. (1995). Protective effect of vanadate on oxyradical-induced changes in isolated perfused heart. Mol. Cell. Biochem..

[87] Baghel S.S., Shrivastava N., Baghel R.S., Agrawal P., Rajput S. (2012). A review of quercetin: Antioxidant and anticancer properties. WJPPS.

[88] Lu L.P., Suo F.Z., Feng Y.L., Song L.L., Li Y., Li Y.J. (2019). Synthesis and biological evaluation of vanadium complexes as novel anti-tumor agents. Eur. J. Med. Chem..

[89] Rauf A., Imran M., Khan I.A., Ur-Rehman M., Gilani S.A., Mehmood Z., Mubarak M.S. (2018). Anticancer potential of quercetin: A comprehensive review. Phytother. Res..

[90] Dhanya R., Arya A.D., Nisha P., Jayamurthy P. (2017). Quercetin, a lead compound against type 2 diabetes ameliorates glucose uptake via AMPK pathway in skeletal muscle cell line. Front. Pharmacol..

[91] Kemeir M.E.H.A. (2013). The protective effect of vanadium sulphate on ethanol-induced gastric ulcer. Bahrain Med. Bull..

[92] Omayone T.P., Salami A.T., Olopade J.O., Olaleye S.B. (2020). Attenuation of ischemia-reperfusion-induced gastric ulcer by low-dose vanadium in male Wistar rats. Life Sci..

[93] De la Lastra C.A., Martin M.J., Motilva V. (1994). Antiulcer and gastroprotective effects of quercetin: A gross and histologic study. Pharmacology.

[94] Nabavi S.F., Russo G.L., Daglia M., Nabavi S.M. (2015). Role of quercetin as an alternative for obesity treatment: You are what you eat!. Food Chem..

[95] Zhang Z.F., Chen J., Han X., Zhang Y., Liao H.B., Lei R.X., Zhuang Y., Wang Z.F., Li Z., Chen J.C. (2017). Bisperoxovanadium (pyridin-2-squaramide) targets both PTEN and ERK1/2 to confer neuroprotection. Br. J. Pharmacol..

[96] Basu A., Bhattacharjee A., Hajra S., Samanta A., Bhattacharya S. (2016). Ameliorative effect of an oxovanadium(IV) complex against oxidative stress and nephrotoxicity induced by cisplatin. Redox Rep..

[97] Alasmari A.F. (2021). Cardioprotective and nephroprotective effects of quercetin against different toxic agents. Eur. Rev. Med. Pharmacol. Sci..

[98] Bhuiyan S., Fukunaga K. (2009). Cardioprotection by vanadium compounds targeting Akt-mediated signaling. J. Pharmacol. Sci..

